# Protracted Clonal Trajectory of a *JAK2* V617F-Positive Myeloproliferative Neoplasm Developing during Long-Term Remission from Acute Myeloid Leukemia

**DOI:** 10.1155/2018/8713020

**Published:** 2018-05-09

**Authors:** Stephen E. Langabeer, Karl Haslam, Maria Anne Smyth, John Quinn, Philip T. Murphy

**Affiliations:** ^1^Cancer Molecular Diagnostics, St. James's Hospital, Dublin 8, Ireland; ^2^Department of Haematology, Beaumont Hospital, Dublin 9, Ireland

## Abstract

Although transformation of the myeloproliferative neoplasms (MPNs) to acute myeloid leukemia (AML) is well documented, development of an MPN in patients previously treated for, and in remission from, AML is exceedingly rare. A case is described in which a patient was successfully treated for AML and in whom a *JAK2* V617F-positive MPN was diagnosed after seven years in remission. Retrospective evaluation of the *JAK2* V617F detected a low allele burden at AML diagnosis and following one course of induction chemotherapy. This putative chemoresistant clone subsequently expanded over the intervening seven years, resulting in a hematologically overt MPN. As AML relapse has not occurred, the MPN may have arose in a separate initiating cell from that of the AML. Alternatively, both malignancies possibly evolved from a common precursor defined by a predisposition mutation with divergent evolution into MPN through acquisition of the *JAK2* V617F and AML through acquisition of different mutations. This case emphasizes the protracted time frame from acquisition of a disease-driving mutation to overt MPN and further underscores the clonal complexity in MPN evolution.

## 1. Introduction

The clonal evolution of a myeloproliferative neoplasm (MPN) into acute myeloid leukemia (AML) is complex: both the *JAK2* V617F and *CALR* exon nine mutations are absent in a significant number of MPN patients evolving to AML implicating the transformation of a common myeloid progenitor at a stage before acquisition of the MPN driver mutation or evolution from a distinct initiating precursor from that harboring the *JAK2* V617F or *CALR* mutation [1–3]. Furthermore, not only the type of additional mutations but also the order in which these mutations are acquired can influence clonal evolution and phenotype [4]. In contrast, the emergence of a *JAK2* V617F-positive MPN on the background of AML is exceedingly rare. Nearly all those cases reported have occurred in AML patients in long-term remission after chemotherapy or autologous stem cell transplantation and where retrospective genotyping of the AML presentation material was performed, demonstrating the absence of the *JAK2* V617F mutation [5–8]. Molecular investigation of another case suggested that the MPN may be secondary to the AML [9]. A case is described of a patient developing a *JAK2* V617F-positive MPN when in remission of AML for seven years and in whom retrospective molecular investigation was able to unravel the evolutionary trajectory of the MPN.

## 2. Case Report

**Figure 1:**
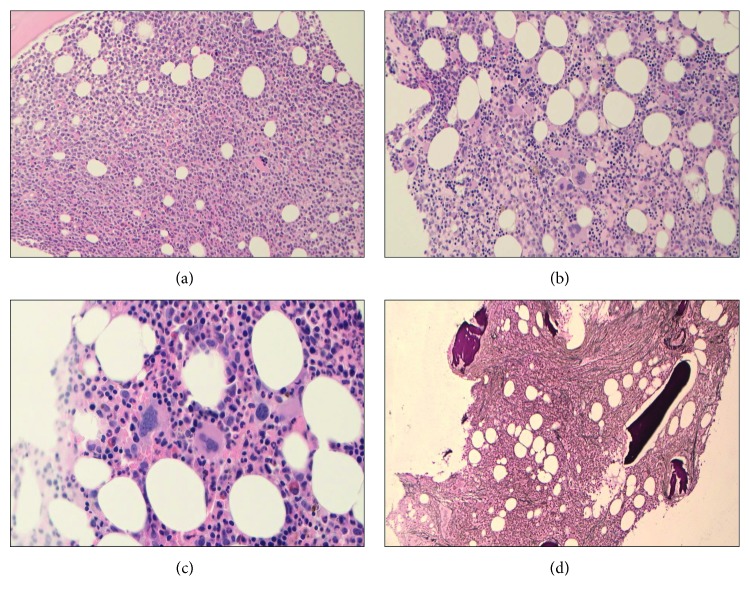
Bone marrow biopsy (a) at diagnosis of acute myeloid leukemia (AML) demonstrating infiltration by myeloblasts; (b) after one course of AML therapy showing atypical megakaryocyte morphology and focal clustering; (c) at diagnosis of myeloproliferative neoplasm (MPN) with myeloid hypercellularity and increased megakaryocytes; and (d) at diagnosis of MPN demonstrating increased reticulin deposition.

A 64-year-old female presented in April 2010 with a two-week history of lethargy. A full blood count revealed a hemoglobin count of 8.6 g/dL, a platelet count of 48 × 10^9^/L, and a white cell count of 96.4 × 10^9^/L with a large population of blasts. On physical examination, there was no palpable liver or spleen. Peripheral blood immunophenotyping showed two blast populations: one population (20%) positive for HLA-DR, CD117, CD33, and CD13 and a second population (48%) positive for HLA-DR, CD33, CD13, CD14, and CD15. The bone marrow aspirate was hypercellular with the vast majority of cells either myeloblasts or monoblasts, all consistent with a diagnosis of acute myeloid leukemia (AML) of myelomonocytic type (Figure 1(a)). Cytogenetic analysis demonstrated a normal karyotype, and there was no evidence of an *FLT3* internal tandem duplication mutation. The patient achieved a morphological remission after one course of daunorubicin and cytarabine (3 + 10). The bone marrow trephine biopsy at this time showed no evidence of blasts but remained hypercellular with some abnormal megakaryocyte forms showing focal clustering (Figure 1(b)). The reticulin stain was normal. The patient completed three courses of consolidation chemotherapy ending in August 2010 with hemoglobin, hematocrit, red cell count, and platelets coming into normal range within two years of finishing AML treatment and remains in remission.

A mild neutrophilia (7.85 × 10^9^/L) was noted in October 2013 and an eosinophilia (0.4 × 10^9^/L) was noted in January 2017, slowly rising to 18.9 × 10^9^/L neutrophils and 0.6 × 10^9^/L eosinophils. Qualitative molecular analysis of peripheral blood in July 2017 showed no evidence of *BCR-ABL1* transcripts but detected the presence of the *JAK2* V617F mutation. Subsequent bone marrow aspirate and biopsy in August 2017 (seven years after completion of AML therapy) revealed hypercellularity with increased myelopoiesis undergoing normal maturation. Megakaryocytes were increased in number with some immature forms being seen and an increase in mature myeloid cells to erythroid cells (Figure 1(c)). Staining for reticulin demonstrated increased deposition (Figure 1(d)), resulting in a diagnosis of MPN unclassified. Currently, the patient is asymptomatic and is on no active therapy to control her white cell count.

Quantitative assessment of the *JAK2* V617F allele burden was performed on the archival material using an assay previously described with a sensitivity of 0.01% mutant alleles [10]. Quantitative PCR demonstrated bone marrow allele burdens of 0.2% at AML diagnosis (in independent duplicate samples), 5.7% after one course of AML therapy, and 72.7% at MPN diagnosis.

## 3. Discussion

**Figure 2:**
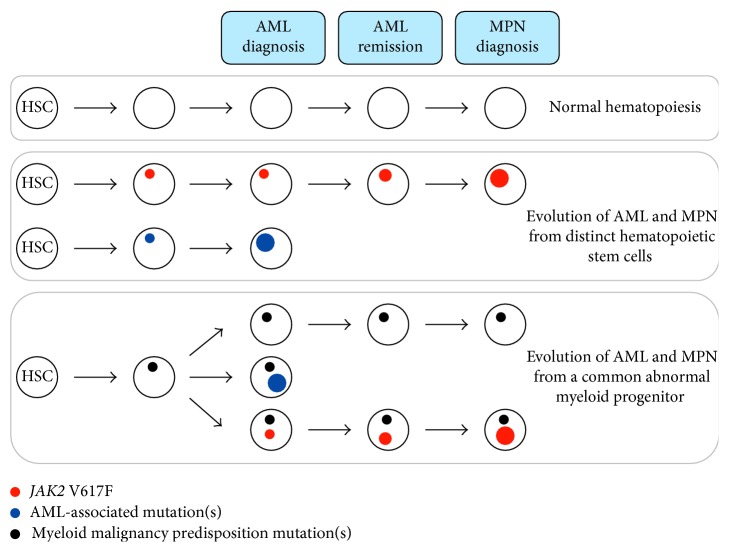
Possible divergent clonal trajectories of the *JAK2* V617F-positive myeloproliferative neoplasm and acute myeloid leukemia in tandem with normal hematopoiesis. AML: acute myeloid leukemia; MPN: myeloproliferative neoplasm; HSC: hematopoietic stem cell.

Molecular analysis of AML derived from a *JAK2* V617F-positive MPN has demonstrated divergent pathways to transformation, highlighting the underlying clonal complexity of MPN evolution. In the case described herein, retrospective molecular investigation detected a low *JAK2* V617F allele burden at AML diagnosis that was further unmasked by the AML induction chemotherapy. As AML relapse has not occurred, the MPN may have arose in a separate initiating cell from that of the AML. Alternatively, both malignancies possibly evolved from a common precursor defined by a predisposition mutation with divergent evolution into MPN or AML through acquisition of the *JAK2* V617F or AML-associated mutations, respectively (Figure 2). The chemoresistant *JAK2* V617F clone [11] remained clinically silent for seven years, although continually expanding, until hematological and morphological diagnosis of an MPN with a high *JAK2* V617F allele burden. The high *JAK2* V617F allele burden likely represents loss of heterozygosity in at least some mutant clones, analogous to that observed in the progression of polycythemia vera [12]. This phenomenon of a minor *JAK2* V617F clone at AML diagnosis has been described previously in a patient who developed polycythemia vera after five years in remission for AML treatment and in whom retrospective analysis revealed a *JAK2* V617F allele burden of 2.0% at the time of AML diagnosis [13]. Whole-exome sequencing and cluster analysis in a recently reported similar case suggest that preleukemic hematopoietic stem cells of AML can not only contribute to myeloid hematopoiesis during remission but may also give rise to another malignancy such as MPN [14]. Moreover, although de novo *JAK2* V617F-positive AML is uncommon, it can be associated with the *t*(8;21) and trisomy 8 cytogenetic abnormalities, with myelodysplastic changes, and a high allele burden [15, 16], features not observed in this case.

A protracted time course is clearly evident from acquisition of the initial *JAK2* V617F driver mutation to development of a hematologically and clinically overt MPN accentuating the different kinetics of development of MPN and AML and suggesting that initiation of the MPN may have been an earlier event relative to that of the AML. Studies have shown that this process may take several years and have attempted to quantify the annual increase in *JAK2* V617F allele burden [17]. A more recent study of long-term follow-up of blood donors who developed an MPN has shown a similar latency though individual variation does exist, which is possibly a reflection of both inherited and acquired genetic factors [18].

Finally, given the expanding focus in minimal residual disease-directed therapy for AML patients [19], caution is warranted in the interpretation of low-level mutations such as the *JAK2* V617F, as these may not represent residual disease but a latent, coexisting hematological malignancy.
